# Rationale and design of the multi organ inflammation with serial testing study: a comprehensive assessment of functional and structural abnormalities in patients with recovered COVID-19

**DOI:** 10.3389/fmed.2024.1392169

**Published:** 2024-07-24

**Authors:** D. Ian Paterson, James A. White, Christian Beaulieu, Rachel Sherrington, Carla M. Prado, Puneeta Tandon, Kieran Halloran, Stephanie Smith, Jennifer A. McCombe, Bruce Ritchie, Edith Pituskin, Mark J. Haykowsky, Richard Coulden, Derek Emery, Albert K. Tsui, Kai Y. Wu, Gavin Y. Oudit, Justin A. Ezekowitz, Richard B. Thompson

**Affiliations:** ^1^University of Ottawa Heart Institute, University of Ottawa, Ottawa, ON, Canada; ^2^Libin Cardiovascular Institute, University of Calgary, Calgary, AB, Canada; ^3^Department of Biomedical Engineering, University of Alberta, Edmonton, AB, Canada; ^4^Department of Radiology and Diagnostic Imaging, University of Alberta, Edmonton, AB, Canada; ^5^Department of Agricultural, Food and Nutritional Science, Faculty of Agricultural, Life and Environmental Sciences, University of Alberta, Edmonton, AB, Canada; ^6^Department of Medicine, University of Alberta, Edmonton, AB, Canada; ^7^College of Health Sciences, University of Alberta, Edmonton, AB, Canada; ^8^Department of Laboratory Medicine and Pathology, University of Alberta, Edmonton, AB, Canada; ^9^Mazankowski Alberta Heart Institute, Edmonton, AB, Canada

**Keywords:** recovered COVID-19, long COVID, MRI, circulating biomarkers, functional assessment

## Abstract

**Introduction:**

Short-term clinical outcomes from SARS-CoV-2 infection are generally favorable. However, 15–20% of patients report persistent symptoms of at least 12 weeks duration, often referred to as long COVID. Population studies have also demonstrated an increased risk of incident diabetes and cardiovascular disease at 12 months following infection. While imaging studies have identified multi-organ injury patterns in patients with recovered COVID-19, their respective contributions to the disability and morbidity of long COVID is unclear.

**Methods:**

A multicenter, observational study of 215 vaccine-naïve patients with clinically recovered COVID-19, studied at 3–6 months following infection, and 133 healthy volunteers without prior SARS-CoV-2 infection. Patients with recovered COVID-19 were screened for long COVID related symptoms and their impact on daily living. Multi-organ, multi-parametric magnetic resonance imaging (MRI) and circulating biomarkers were acquired to document sub-clinical organ pathology. All participants underwent pulmonary function, aerobic endurance (6 min walk test), cognition testing and olfaction assessment. Clinical outcomes were collected up to 1 year from infection. The primary objective of this study is to identify associations between organ injury and disability in patients with long-COVID symptoms in comparison to controls. As a secondary objective, imaging and circulating biomarkers with the potential to exacerbate cardiovascular health were characterized.

**Discussion:**

Long-term sequelae of COVID-19 are common and can result in significant disability and cardiometabolic disease. The overall goal of this project is to identify novel targets for the treatment of long COVID including mitigating the risk of incident cardiovascular disease.

**Study registration:**

clinicaltrials.gov (MOIST late cross-sectional study; NCT04525404).

## Introduction

The coronavirus disease (COVID)-19 pandemic has caused significant worldwide death and disability, particularly prior to the widespread availability of vaccine. Since the start of the pandemic, there have been seven waves of COVID-19 caused by evolving variants of the Severe Acute Respiratory Syndrome coronavirus-2 (SARS-CoV-2) (1, 2). In Canada, the province of Alberta, a region with 4.5 million inhabitants, has consistently had the highest seroprevalence for SARS-CoV-2 and in November 2023 approximately 85% had evidence for prior infection (3). However, the immediate health risk from acute infection has been low with a hospitalization rate of 5.5% and death in 1% (4).

While the short-term prognosis of COVID-19 is excellent, the intermediate and long-term health risks are of greater concern. A 2022 national survey found that approximately 17% of Canadians with COVID-19 report persistent symptoms lasting greater than 12 weeks, a syndrome referred to as long COVID or post COVID condition (5, 6). Symptoms are often characterized by fatigue, shortness of breath and/or cognitive impairment, with a disproportionate effect in women (5). Among affected individuals, 47% reported symptoms lasting at least 1 year and 21% described symptoms that often or always limited daily activities. The national survey also found that 27% of patient with SARS-CoV-2 developed long COVID following the Alpha variant infection compared to 13% with the Omicron variant (5).

Furthermore, population health studies have shown that COVID-19 confers a 50–70% excess risk of incident cardiovascular disease and diabetes mellitus in the first 12 months (7, 8). These health risks appear greater in patients with long COVID (9). The mechanism(s) responsible for long COVID and the increased cardiometabolic risk are not well understood and there is a lack of high-quality evidence-based studies guiding management.

The prevailing etiologic mechanisms proposed for long COVID include immune dysregulation, autoimmunity and immune imprinting, endothelial dysfunction and thrombosis, impaired neurological signaling and effects on the host microbiome (10). However, knowledge on pathogenesis remains limited and there is an important unmet need for rigorous preclinical and clinical studies in long COVID. Given the systemic (i.e., multi-organ) nature of both acute phase COVID-19 illness and long COVID, knowledge has been gained from whole body imaging. Magnetic resonance imaging (MRI) is a safe (non-ionizing radiation), non-invasive imaging technique that provides detailed information on tissue changes including injury. MRI has been used as an alternative to computed tomography for the characterization of pulmonary disease following severe COVID-19 pneumonia (11). MRI derived measures of visceral adipose tissue and liver fat are also strongly associated with risk of hospitalization from COVID-19 independent of body mass index (12). Whole body MRI-based studies of patients with recovered COVID-19 have identified subclinical multi-organ involvement (13–15). In an MRI study of 201 patients with long COVID, mean age 45 years, organ damage was identified in 70%, including the pancreas in 45%, liver in 29% and heart in 24% (14). In this study, MRI evidence of organ impairment was defined as a non-contrast T1 time (longitudinal relaxation time) greater than normal reference values. Several cardiac MRI based studies of patients with recovered COVID-19 have found evidence of subclinical myocardial inflammation (16–18), however, the clinical significance of this finding is not well established.

We undertook a multicenter, prospective study of patients in Alberta with recovered COVID-19 from October 2020 to August 2021 to characterize symptom burden, functional impairment and end-organ damage by MRI. We hypothesized that the extent of tissue injury on MRI would be associated with patient reported disability and objective measures of functional performance.

Our primary objective was to comprehensively apply multi-system MRI to assess the presence and extent of organ injury (heart, lungs, brain, abdominal viscera and skeletal muscle) among patients with recovered COVID-19 and compare these findings between patients with moderate to severe symptoms, minimal symptoms, and healthy controls without prior infection. As a secondary objective, we also sought to characterize imaging and circulating biomarkers with the potential to exacerbate cardiovascular health. Additionally, we incorporated opportunistic supplementary studies to explore the impact of COVID-19 on energy metabolism and patient’s perspectives.

## Methods and analysis

### Study design

This is an observational prospective case–control study of patients in Alberta with recovered COVID-19 from the first 2 waves of the pandemic and age- and sex-matched healthy control participants without prior COVID-19 infection. Institutional approval was obtained from the health research ethics boards at the University of Alberta for the study of patients with recovered COVID-19 (Pro00102389), the two supplementary studies (Pro00109391, Pro00110221) and the healthy controls (Pro00110706) and at the University of Calgary for patients with recovered COVID-19 (REB21-0035). The study was also registered at clinicaltrials.gov (MOIST late cross-sectional study; NCT04525404). All patients provided written informed consent. The Canadian VIGOUR Centre (thecvc.ca) helped provide project management, and the study team vouches for the data integrity and analyses of the study. Patient related variables were captured in REDCap (Research Electronic Data Capture) hosted at the University of Alberta and imaging data was stored in a secure server in the department of biomedical engineering at the University of Alberta. The principal investigator (DIP) oversaw site monitoring and data management and will supervise all analyses related to the study.

### Participant selection

Adult patients within 3 months of COVID-19 illness and prior to the availability of m-RNA vaccination, were recruited from October 2020 to July 2021. Patients with COVID-19 illness requiring hospitalization were identified prospectively at the University of Alberta hospital and retrospectively by the regional health authority, Alberta Health Services. Interested patients with less severe COVID-19 illness also contacted the study team following advertisement on the internet, mainstream media and personal communication. All patients required documentation of COVID-19 infection on nasal or oropharyngeal swab polymerase chain reaction testing within the last 6 months. Healthy controls without prior COVID-19 infection were also recruited from July 2021 to July 2023. Normative brain imaging and cognitive testing were collected separately from healthy control participants prior to the pandemic (19). Control participants with a history of cardiovascular disease or cardiovascular risk factors were excluded. No participants had contraindication to MRI and all provided informed consent after a review of the study objectives, procedures and potential risks and benefits.

### Data collection and analysis

#### Medical profile, post-COVID-19 symptoms, and blood collection

**Figure 1:**
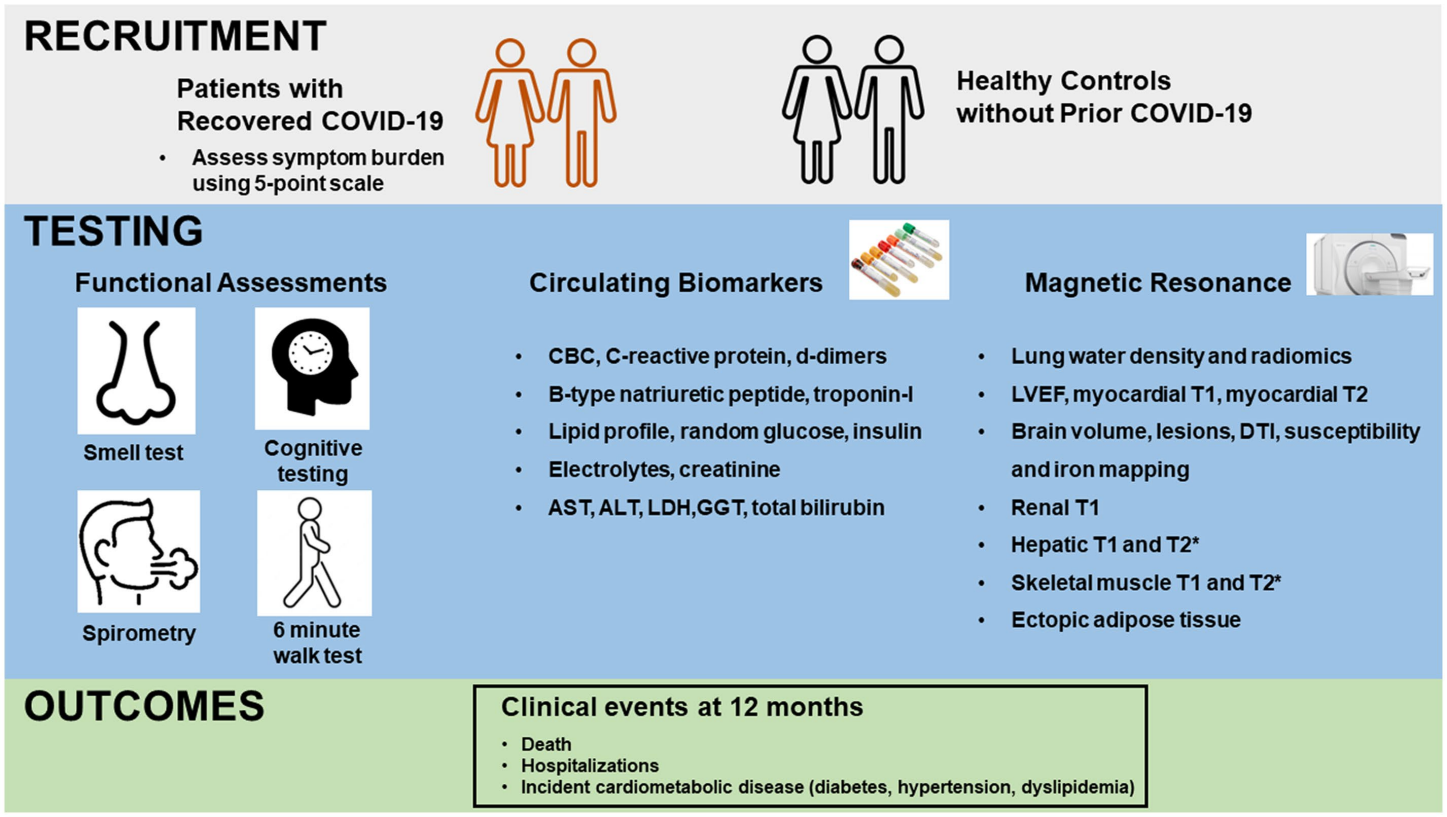
Schema of study design. Upper panel depicts recruitment of patients with recovered COVID-19 and healthy controls without prior COVID-19. Middle panel depicts functional testing, blood collection and magnetic resonance imaging for all participants. Functional testing included Brief Smell Identification Testing (B-SIT), 6 min walk testing, cognitive testing on a tablet with NIH toolbox and spirometry. Bottom panel depicts collection of clinical outcomes at 12 months. CBC, complete blood count; AST, aspartate aminotransferase; ALT, alanine aminotransferase; LDH, lactate dehydrogenase; GGT, gamma-glutamyl transferase; LVEF, left ventricular ejection fraction; DTI, diffusion tensor imaging.

Participants were scheduled for same day comprehensive testing inclusive of a medical review, functional performance, blood collection and imaging (Figure 1).

A detailed assessment of relevant medical history and medication use was achieved through direct questioning and a review of health records. In patients with recovered COVID-19, data on the timing and duration of illness, the need for hospitalization and transfer to intensive care were recorded.

At the time of study-related functional assessments and MRI, patients with recovered COVID-19 were screened for the presence of long COVID related symptoms including fatigue, cognitive impairment, shortness of breath, chest pain and palpitations using a standardized questionnaire. Additionally, patients rated the overall impact of these symptoms on their daily activities using a 5-point scale ranging from 1 (no limitations) to 5 (severely limited).

Blood pressure, heart rate and oxygen saturation were measured on all participants and blood work was performed to assess for circulating biomarkers of inflammation (c-reactive protein, white blood cell count with differential and d-dimer), end organ damage (high sensitivity cardiac troponin I, b-type natriuretic peptide, creatinine and hepatic enzymes) and cardiometabolic profile (glucose, insulin and lipid profile). Blood biospecimens were also stored in a research biobank (Canadian BioSample Repository) for future analysis.

### Functional assessments

Standardized testing to evaluate olfaction, cognition, lung function and functional capacity was administered by trained research personnel. Smell was evaluated using the Brief Smell Identification Test (BSIT) which requires the identification of 12 odors from a scratchable booklet. In healthy older individuals, impaired olfaction on BSIT predicts cognitive decline (20). Cognitive performance was ascertained during 30–40 min sessions from the NIH toolbox with patients completing 5 modules including the Picture Sequence Memory Test, List Sorting Working Memory Test, Dimensional Change Card Sort Test, Auditory Verbal Learning Test and Oral Symbol Digit Test. Hand-held spirometry measured forced expiratory volume in 1 s and forced vital capacity during 3 repetitions. Six-minute walk test (6MWT) was performed to assess aerobic endurance using standardized instructions (21). Patients also performed a 25-foot timed walk test to identify potential neurologic disease affecting mobility (22).

### Magnetic resonance imaging

**Figure 2:**
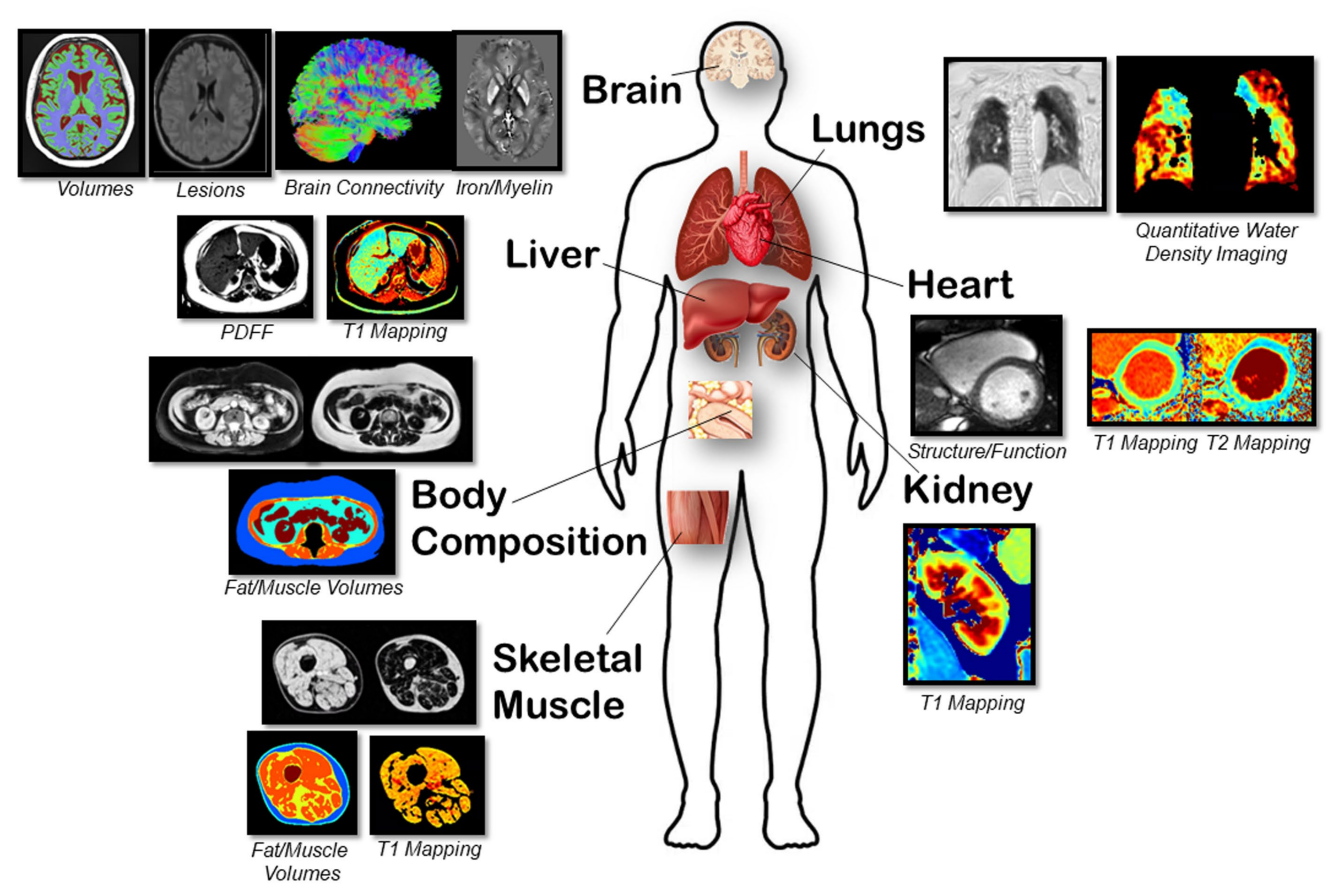
Representative magnetic resonance images of multiple organs are shown for. Brain: Volumes with T1, lesions with FLAIR, white matter connectivity with DTI, and iron/myelin indication with QSM/R2* sequences. Lungs: Parenchyma lung water density quantification using free-breathing yarnball sequence. Heart: Structure, function from cine imaging and T1 and T2 mapping sequences. Body Composition: Abdominal fat/water separated imaging with chemical-shift encoded approach (multi-echo gradient echo sequence). Liver: PDFF, water-specific T1 and T2* using a SR-CSE sequence. Kidney: T1 mapping using the MOLLI sequence. Skeletal Muscle: PDFF, water-specific T1 and T2* with calculation of fat and muscle volumes and muscle T1.

A multiparametric, non-contrast research MRI was performed at 3 T (Magnetom Prisma, Siemens Healthineers) at the Universities of Alberta and Calgary with a total scan time of approximately 75 min (Figure 2). Image analysis for cardiac and non-cardiac data was performed at core lab facilities within the University of Calgary and University of Alberta, respectively.

*Cardiac*: Standard imaging sequences were used to assess cardiac structure and function. Steady-state free precession cine imaging was acquired with retrospective ECG gating with full left and right ventricular coverage. Typical acquisition parameters were 1.09 ms echo time, 2.53 ms repetition time, 30° flip angle, 8 mm slice thickness with a 2 mm gap, field of view 400 × 300 mm, acquisition matrix 256 × 144, 1,500 Hz/pixel, 15 views per segment, rate 3 parallel imaging (GRAPPA), and 30 reconstructed cardiac phases. Native myocardial T1 mapping was acquired using the modified Look-Locker inversion recovery (MOLLI) sequence (23) from a single mid ventricular short axis slice with typical parameters: 0.89 ms echo time, 2.47 ms repetition time, 35° flip angle, 8 mm slice thickness, field of view 410 × 330 mm, acquisition matrix 224 × 140, 1,395 Hz/pixel, rate 2 parallel imaging (GRAPPA), 5(3)3 protocol. T2 mapping was acquired with matching slice location, field of view, resolution and flip angle with a 1.19 ms echo time and 2.77 ms repetition time.

Ventricular volumes, mass, ejection fraction, global longitudinal strain and myocardial T1 will be derived using commercially available software (cvi42, Version 5.13, Circle, Calgary, Canada). Regional myocardial T1 and T2 was measured in 6 equal segments from the mid ventricular slice.

*Body Composition*: A chemical-shift encoded acquisition enabled the generation of fat and water separated images with transverse slice prescriptions centered on the third lumbar vertebra (L3) (24). Typical image parameters included 8 axial slices with a 6 mm thickness, [1.65, 3.61, 5.57, 7.53, 9.49, 11.45] ms echo times, 13.1 ms repetition time, 30° flip angle, rate 2 parallel imaging (GRAPPA) for an 8 s end-expiration breath-hold acquisition with a subset of 3 images selected for analysis (25). For the body composition analysis, the volumes of skeletal muscle, intermuscular, visceral, and subcutaneous fat from contiguous axial slices will be measured using custom fully automated machine learning segmentation. Muscle and fat volumes from a cross-section at L3 have been shown to be accurate relative to cadaver measurements and representative of whole-body composition (26, 27).

*Liver*: Liver T1 and proton density fat fraction (PDFF) was acquired using a water-specific T1 mapping approach to eliminate systematic T1 errors from liver fat using a saturation-recovery chemical-shift encoded (SR-CSE) technique (28–30). Typical image parameters include 3 axial slices with a 6 mm thickness, [1.09, 2.45, 3.81, 5.17, 6.53, 7.89] ms echo times, 9.2 ms repetition time, 13° flip angle, rate 2 parallel imaging (GRAPPA) for a 6 s breath-hold acquisition. The liver will be manually traced on all three slices using custom software, with automated removal of blood vessels, with calculation of median T1, PDFF and T2* values as previously described (30).

*Kidney*: T1 mapping of the kidneys was acquired using the MOLLI sequence from a single coronal slice prescribed through the maximum cross-sectional area of both kidneys. Typical acquisition parameters: 0.98 ms echo time, 2.63 ms repetition time, 35° flip angle, 6 mm slice thickness, field of view 450 × 330 mm, acquisition matrix 224 × 144, 1,395 Hz/pixel, rate 2 parallel imaging (GRAPPA), 5(3)3 protocol. Custom software will be used to trace a line along the length of the renal cortex to select intersecting pixels, and circular regions of interest will be selected in renal medulla.

*Skeletal Muscle*: Skeletal muscle T1 and fat content (intermuscular, intramuscular, and subcutaneous) was measured using a muscle-specific variant of the SR-CSE approach. Typical image parameters include 5 axial slices (centered 17 cm superior to the distal head of the femur) with a 3.5 mm thickness (12.5 mm gap), [2.51 3.51 4.51 4.78 5.78 6.78] ms echo times, 9.0 ms repetition time, 30° flip angle, rate 2 parallel imaging (GRAPPA) for a 41 s acquisition. A custom machine learning segmentation approach will be used to identify subcutaneous fat, intermuscular fat, muscle and bone regions. Calculated parameters included volumes of subcutaneous fat, muscle, intermuscular fat, intramuscular fat (fat content in the muscle region), muscle T1 and muscle T2* (from the muscle region).

*Lungs*: Lung images were acquired using a custom non-Cartesian ultrashort TE (TE = 70 μs) yarnball *k*-space trajectory with free-breathing acquisitions (31). Free-breathing data collection was completed in 120 s with reconstruction of 2 mm × 2 mm × 2 mm resolution images at 20 respiratory phases over the breathing cycle. Global lung water density at functional residual capacity (minimum lung volume) was quantified with a user-independent machine learning lung segmentation approach. Additionally, the presence of patchy pathology was identified using an automated quantitative approach employing radiomic analysis of the lung parenchyma. We used a previously trained deep learning model to segment the lung parenchyma of our images (32). Once the parenchyma of each lung is isolated, intensities are discretized into 5% lung water density bins to simplify image features prior to feature extraction. Finally, 40 radiomic texture features are computed in three dimensions for each lung using a MATLAB (MathWorks, Natick, MA) toolkit (33).

*Brain*: After repositioning the participant into a 64 channel head RF coil, four different images were acquired of the brain over 24 min: (i) 3D fluid attenuation inversion recovery (FLAIR) for lesion detection (1.0 mm isotropic, 5 min), (ii) 3D T1-weighted MPRAGE for regional brain volumes (0.85 mm isotropic, 3.5 min), (iii) 2D high-resolution diffusion tensor imaging (DTI) for identifying strokes/cytotoxic edema and microstructure in white matter mainly (1.5 mm isotropic, 10 b0/6 b500/20 b1000/64 b2500 s/mm^2^, 9.5 min), and 3D multi-echo, gradient recalled echo (0.9 × 0.9 × 1.7 mm^3^, 6 TE from 3.8–31 ms, 5.5 min) for quantitative susceptibility mapping (QSM) and transverse relaxation rate (R2*) for micro-bleeds and sensitivity to iron/myelin, particularly in deep gray matter. The latter three images match previous protocols of a healthy cohort (19).

### Study outcomes

Primary outcomes include patient reported symptom burden and organ injury metrics as assessed by functional performance evaluations, serum biomarkers and MRI tissue characterization (Table 1). Secondary outcomes include imaging and circulating biomarkers of cardiovascular health (Table 2). Clinical events at 12 months will be collected though chart review to ascertain relevant clinical outcomes including incident diabetes and cardiovascular disease.

**Table 1:** Summary of organ injury outcomes.

Organ	Functional performance	Circulating biomarkers	Magnetic resonance
LUNG	Spirometry 6 min walk testing	None	Lung water density Lung lesions (radiomic analysis)
CARDIAC	6 min walk testing	Troponin I B-type natriuretic peptide	LVEF Myocardial T1 Myocardial T2
BRAIN	Cognitive testing	None	Brain volume Brain lesions (FLAIR and DTI) Susceptibility and iron mapping
HEPATIC	None	AST ALT GGT LDH Total bilirubin	Hepatic T1 Hepatic T2*
RENAL	None	Electrolytes Creatinine	Renal T1
SKELETAL MUSCLE	6 min walk testing	None	Skeletal muscle T1 Skeletal muscle T2*

AST, aspartate aminotransferase; ALT, alanine aminotransferase; LDH, lactate dehydrogenase; GGT, gamma-glutamyl transferase; LVEF, left ventricular ejection fraction; FLAIR, fluid attenuation inversion recovery; DTI, diffusion tensor imaging.

**Table 2:** Summary of cardiovascular health outcomes.

	Circulating biomarkers	Magnetic resonance	Clinical events
CARDIAC	Troponin I B-type natriuretic peptide	LVEF Myocardial T1 Myocardial T2	Cardiovascular death or hospitalization
CARDIOMETABOLIC	C-reactive protein White blood cell count Glucose Insulin Lipid profile	Visceral adipose tissue Hepatic fat Intramuscular fat Intermuscular fat	Incident diabetes Incident dyslipidemia Incident hypertension

LVEF, left ventricular ejection fraction.

### Statistical plan

The Shapiro–Wilk normality test will be used to test the normal distribution of continuous variables which will be expressed as mean ± standard deviation or median (25th, 75th percentile), as appropriate. Categorical variables will be expressed as frequency and percentage. When comparing data between recovered COVID-19 patients and healthy controls, chi-square testing or Fisher’s exact test will be used for categorical variables and two sample t-test or Mann–Whitney U test used for continuous variables, as appropriate. Functional performance, biomarker results and imaging metrics indicative of organ injury will be compared across groups of symptom severity (none, mild, moderate and severe) within the recovered COVID-19 patients using a one-way analysis of variance with *post hoc* pairwise comparisons by either Tukey’s or Games-Howell tests, depending on equality of variances. Pearson correlation analyses will evaluate relationships between MRI measures of tissue injury and functional performance metrics. Univariable Cox proportional regression of clinical outcome will be performed in all serum and imaging parameters. In the multivariable Cox proportional hazard analysis, all non-collinear parameters of interest with univariable *p*-value < 0.2 will independently test for their association with composite outcome after adjustment for baseline risk. A *p* value less than 0.05 will be considered significant for all tests.

### Sample size calculation

There is little data informing on associations between symptom burden, functional performance and MRI derived tissue composition in patients with recovered COVID-19. At the time of our study conception, only one MRI study reported on 100 patients at a median of 71 days from COVID-19 illness and found increased native myocardial T1 compared to 50 healthy controls, median 1,125 ms vs. 1,082 ms, respectively. They also reported increase myocardial T1 in those with prior hospitalization (*N* = 33) compared to patients who had recovered at home (*N* = 67), median 1,141 ms vs. 1,119 ms, *p* = 0.008 (16). A subsequent multicenter study of 148 patients with prior hospitalization for severe COVID-19 also found increased myocardial T1 compared to 40 healthy volunteers, mean 1,033 ms vs. 1,008 ms, *p* < 0.001 (17). However, data linking imaging to symptoms and functional performance is inconclusive. In a multi-organ, MRI based study of 201 patients with long COVID, only abnormalities in myocardial T1 were associated with severe symptom burden and/or disability (14). A multisystem, MRI-based study of 54 patients with prior hospitalization for severe COVID-19 found that imaging derived organ injury (cardiac, renal and hepatic T1 and brain T2*) was associated with circulating biomarkers of inflammation but not with functional performance (spirometry, 6MWT and cognitive testing) (13). Given the lack of data on potential associations of MRI tissue characterization with symptom burden and/or functional performance at the time, we empirically established a target of 200 patients with recovered COVID-19 and 100 age- and sex-matched healthy controls without prior infection. Clinical characteristics of the study population are presented in Table 3.

**Table 3:** Clinical characteristics.

	Recovered COVID-19	Healthy controls
Number of patients	215	133
Female, (%)	139 (65%)	68 (51%)
Age	51 (14)	47 (15)
Caucasian race, (%)	170 (79%)	113 (85%)
Height, cm	168 (10)	170 (9)
Weight, kg	83 (20)	72 (13)
Systolic blood pressure, mmHg	134 (18)	122 (13)
Diastolic blood pressure, mmHg	84 (11)	80 (9)
Heart rate, bpm	71 (12)	73 (13)
Oxygen saturation (%)	98 (2)	98 (2)
**MEDICAL HISTORY**		
Current smoker, (%)	9 (4%)	1 (1%)
Past smoker, (%)	50 (23%)	8 (6%)
Alcoholic beverages/week	1.8 (2.6)	3 (4)
Hypertension, (%)	51 (24%)	0
Diabetes mellitus, (%)	28 (13%)	0
Dyslipidemia, (%)	38 (18%)	0
Alcohol overuse, (%)	2 (1%)	0
Coronary artery disease, (%)	4 (2%)	0
Heart failure, (%)	1 (0.5%)	0
Atrial fibrillation (%)	4 (2%)	0
COPD, (%)	23 (11%)	0
Sleep apnea, (%)	14 (7%)	0
Stroke, (%)	5 (2%)	0
Cognitive impairment, (%)	3 (1%)	0
Neuropathy, (%)	7 (3%)	0
Renal insufficiency, (%)	7 (3%)	0
Liver disease, (%)	0	0
Prior cancer, (%)	17 (8%)	0
Pneumonia in last year, (%)	2 (1%)	0
**COVID-19 Illness**		
Duration of Illness		
1–7 days, (%)	80 (37%)	Not applicable
8–14 days, (%)	68 (32%)	
>14 days, (%)	67 (31%)	
Hospitalized, (%)	59 (27%)	
Intensive care unit, (%)	17 (8%)	
Ventilation, (%)	11 (5%)	

Values are represented as total number (%) or mean (standard deviation).

### Supplementary studies

Between May and October 2021, patients from the recovered COVID-19 group were contacted via phone and email and invited to participate in supplementary studies of energy metabolism and patient perspectives. Based on emerging data and observations, these sub-studies were opportunistically conceptualized after the primary study had been initiated. In the supplementary study on energy metabolism, a subset of patients were examined to investigate the role of the most metabolically active organs affecting resting energy expenditure (REE). Notably, organs significantly influence REE, accounting for approximately 75% of this energy metabolism component. Given the systemic effects of COVID-19, understanding potential changes in energy metabolism post-recovery is imperative due to its impact on body composition and nutritional status in general (34–36). By examining the impact of individual organs on REE in those recovered from COVID-19, we aim to gain insights into the lasting metabolic effects of infection, distinct from its acute complications. Knowledge gained could optimize health in the post-recovery phase by guiding targeted interventions. Exclusion criteria for this supplementary study included pregnancy or lactation, having any electronic implant and those who are claustrophobic. Specific protocols were followed for REE assessment as described previously (37). Participant’s REE were assessed using a metabolic cart with ventilated hood system (Vmax^®^ Series, CareFusion, Yorba Linda, CA, United States) at the Human Nutrition Research Unit (University of Alberta, Edmonton, AB, Canada).

Abnormalities in energy metabolism will be explored compared to commonly used equations. A new approach will be tested to evaluate the resting metabolic rate *K*(*i*) values of major organs (liver, heart, lungs, kidneys and brain) and tissues on the basis of a mechanistic model: REE = Σ(*K*(*i*) × *T*(*i*)), where REE is whole-body REE measured by indirect calorimetry, and *T*(*i*) is the mass of individual organs and tissues measured by MRI. With measured REE and *T*(*i*), marginal 95% confidence interval for *K*(*i*) values will be calculated by stepwise univariate regression analysis (38).

For the supplementary study on patient experiences, we sought to learn about the individual experience of acute and recovered COVID-19 and to determine if patient reported symptoms and experiences correlated to physiologic testing. An audio-taped 45-to-60-min interview was conducted, using an open-ended style. The researcher recorded field notes after each interview, including general observations, any important nonverbal communication, and thoughts or feelings regarding the interview *(“memoing”).* The data collected was transcribed verbatim and stripped of potential identifiable material by a professional transcription company.

For the analysis, a broad-based data coding system will be created and considered in contrast to other groupings with different properties. These initial codes will then be developed into concepts, themes, and potential sub-themes, into what is termed “pattern recognition.” The final synthesis of the data will be achieved when the researcher has reached a level of interpretation that develops a conceptual definition that will be meaningful and relevant to applied practice.

## Discussion

The primary goal of this study is to improve knowledge of the mechanisms governing disability and poor health in patients with persistent COVID-19 symptoms. We have therefore undertaken a comprehensive multiparametric assessment of post-COVID-19 sequelae in a large cohort of survivors and healthy controls. Novel aspects include a comprehensive MRI-based characterization of body composition and detailed measures of functional impairment. The results from this study could inform on potential therapeutic targets for long COVID syndrome including post-COVID related cardiovascular risk. Long COVID continues to affect approximately 10% of patients infected with the Omicron variant (5, 39). Therefore, these results could inform on current practice and provide justification for clinical trials of long COVID.

### Ethics and dissemination

Given the observational nature of this study, ethical and safety concerns are minimal. Serum samples and imaging data are stored at secure repositories at the University of Alberta. Requests for access to data will be provided upon reasonable request where permissible by institutional governance. Results from this trial will be disseminated through presentation at scientific meetings, manuscript publications, knowledge translation activities with national and international societies and incorporation into clinical guidelines. We will actively engage patient groups through public speaking engagements facilitated by long COVID networks. Long COVID groups are also active on social media and we will disseminate results through these platforms.

## Ethics statement

The studies involving humans were approved by the University of Alberta and the University of Calgary Health Research Ethics Boards. The studies were conducted in accordance with the local legislation and institutional requirements. The participants provided their written informed consent to participate in this study.

## Author contributions

DP: Conceptualization, Data curation, Funding acquisition, Investigation, Methodology, Project administration, Resources, Supervision, Validation, Writing – original draft, Writing – review & editing. JW: Conceptualization, Data curation, Formal analysis, Funding acquisition, Investigation, Methodology, Project administration, Resources, Software, Supervision, Writing – review & editing. CB: Conceptualization, Data curation, Formal analysis, Funding acquisition, Investigation, Methodology, Project administration, Resources, Software, Supervision, Validation, Writing – review & editing. RS: Investigation, Project administration, Writing – review & editing. CP: Conceptualization, Data curation, Formal analysis, Investigation, Methodology, Resources, Software, Supervision, Validation, Writing – review & editing. PT: Investigation, Methodology, Writing – review & editing. KH: Investigation, Methodology, Writing – review & editing. SS: Investigation, Methodology, Validation, Writing – review & editing. JM: Investigation, Methodology, Writing – review & editing. BR: Investigation, Methodology, Resources, Supervision, Writing – review & editing. EP: Data curation, Formal analysis, Investigation, Methodology, Writing – review & editing. MH: Data curation, Formal analysis, Funding acquisition, Investigation, Resources, Supervision, Validation, Writing – review & editing. RC: Formal analysis, Project administration, Resources, Supervision, Writing – review & editing. DE: Project administration, Resources, Supervision, Writing – review & editing. AT: Formal analysis, Investigation, Methodology, Validation, Writing – review & editing. KW: Data curation, Investigation, Supervision, Writing – review & editing. GO: Investigation, Methodology, Resources, Writing – review & editing. JE: Data curation, Investigation, Methodology, Project administration, Resources, Supervision, Validation, Visualization, Writing – review & editing. RT: Data curation, Formal analysis, Funding acquisition, Investigation, Methodology, Project administration, Resources, Software, Supervision, Validation, Visualization, Writing – review & editing.
